# Pathogenesis

**Published:** 1868-09-15

**Authors:** H. Lebert

**Affiliations:** Professor of Clinical Medicine of the University of Breslau


### CHICAGO MEDICAL JOURNAL.

*Vol. XXV.*—*SEPTEMBER* 15, 1868.—*No.* 18.

### PATHOGENESIS.

*The Pathological Anatomy and Pathogenesis of Disseminated Chronic Pneumonia, and of Pulmonary Tubercles.* *By* H. LEBERT, *Professor of Clinical Medicine of the University of Breslau.*

TRANSLATED BY WALTER HAY, M.D., ASSOCIATE EDITOR CHICAGO MEDICAL JOURNAL.

(*Continued from p.* 559.)

THE pulmonary tissue around these inflammatory foci is rarely normal in their immediate proximity; most frequently hyperæmia, carnification, and diffuse lobular pneumonia may be associated with it temporarily. At other times, the diffuse pneumonia has pursued its ordinary progress, but in place of terminating by resolution, it has passed through in the main all these same phases which have just been described under disseminated chronic pneumonia, which ordinarily is then developed at the same time, and the greater part of the time consecutively in other portions of the same or in the other lung. There is thus presented the intermingling of diffuse and disseminated chronic pneumonia.

The pleura participates almost constantly in chronic pneumonia with scattered force, ordinarily with adhesions, thickening rather than with fluid effusion. When these adhesions are solid, old, thick, they produce at once immobility of the corresponding intercostal spaces, and a fibrous transformation propagating itself, step by step, from the surface of the lung into its interior.

It is through these adhesions that the collateral circulation is, in part constituted, supplying the place of the numerous vessels whose disappearance has been occasioned by the chronic pneumonia.

The relation of tubercles with pneumonia has been indicated in a different manner. Some assert that there are almost always tubercles when chronic scattered pneumonia exists; others regard this as consecutive. I have arrived at the opposite conclusion, that disseminated chronic pneumonia exists most frequently alone without tubercles, and when these latter are developed, they are generally of a more recent date, rather consecutive, sequences not consequences of the pneumonia. The greater portion of the cicatrices at the summits of the lungs are of inflammatory, not tuberculous, origin.

As, in general, every prolonged inflammation develops secondary inflammations, so is it with disseminated chronic pneumonias, phlegmasiæ which tend to a process of ulceration and necrosis.

Among the frequent localizations of this kind, ulcers of the larynx, the epiglottis, of the vocal cords, and of the arytenoid region should be mentioned. Ulcers of the trachea are rare, and still more so are those of the bronchi, whose mucous surface is ordinarily the seat of chronic inflammation; however, in the smaller bronchi the process of cavernous ulceration may as well originate from the mucous membrane as attack it secondarily.

Further, the pneumonic foci occasion the disappearance of many of the minute bronchial ramifications, whilst other and more voluminous branches dilate, especially in the upper portions of the lungs. The bronchial glands are ordinarily tumified and exhibit sometimes little hyperplastic (tuberculous?) foci, or more extensive yellowish infiltrations.

When many of the bronchial glands are infiltrated and voluminous, they compress the nerves and neighboring vessels, and thus provoke grave complications. I have likewise, also, observed an hypertrophy, simple but considerable and multiple, of these glands in disseminated chronic pneumonia as well as in pulmonary tuberculosis. When the infiltrated bronchial glands suppurate, the abscesses may open into the pericardium, into the vena-cava superior, into the bronchi, into the æsophagus, into the lungs, and the pleura. On the other hand, it is not rare to find bronchial glands consisting of dessicated infiltration of stony concretions.

The heart is normal, small, with a tendency to fatty degeneration when the marasmus has attained considerable proportions. The digestive tube is rarely healthy. Gastric and intestinal catarrh and especially ulcers of the small intestine and of the colon, even without any intestinal tubercles whatsoever, occur. The mesenteric glands often exhibit the yellowish infiltration of tubercular appearance as the bronchial glands; stony concretions, likewise, especially are found there; suppuration is here rare. The peritoneum becomes inflamed, especially in the vicinity of intestinal ulcers; here are observed inflammatory granulations much resembling tubercles, which are themselves far from being rare in this serous membrane; there are also found here, especially, voluminous yellowish masses, surrounded with much black pigment, sometimes even with a cortical substance, cellulo-vascular, a true inflammatory product, which in its turn has developed by cellular multiplication these compact or soft masses of a dull yellow color. Inflammatory or tuberculous granulations are found also in the filaments, bridles and false ligaments consecutive to peritonitis. Besides the adhesive and plastic character, besides the complication with true tubercle, there are likewise found serous and sero-purulent fluids. Tubercular peritonitis may exist associated with chronic pneumonia, as well as with pulmonary tubercles, just as a peritonitis, not tubercular, may complicate these two kinds of pulmonary alteration. When profound intestinal alterations exist, the liver is ordinarily fat.

Very small miliary tubercles are here very frequently met with in true pulmonary tuberculization, but very rarely in chronic pneumonia. The same is true of the kidneys and the spleen. These three organs may likewise be the seat of amyloid degeneration, and then the intestinal ulcers may have the same origin. This especially is, a very frequent complication, but not a sequel of disseminated chronic pneumonia. Among the cerebral alterations, inflammation of the internal membrane of the ventricles, and acute hydrocephalus are met with in a certain number of cases; tubercular meningitis as well as more voluminous tubercles of the dura mater and of the brain are more frequently consecutive to true tubercle, and sustain scarcely any intimate relation with disseminated chronic pneumonia. In the large tubercular masses of the nervous centres, it is not rare to find a vascular envelope with a connective tissue in process of prolification, a tissue of a reddish yellow, sometimes semi-transparent, which towards the centre of the tubercles becomes dull, dry, and sometimes exhibits softened portions. The kidneys are rarely diseased in chronic pneumonia, but are sometimes the seat of parenchymatous inflammation. Large tuberculous masses with ulceration are here rare.

Tuberculosis of the mucous surface of the genito-urinary organs has this peculiarity, that it constitutes rather an incrustation of the mucous membrane than a submucous deposit.

The testicle, especially the epididimis, may also be the seat of disseminated inflammation, at a later period confluent with a dull, yellow, dry infiltration rather than with true tubercular granulations.

It may be said that generally disseminated chronic pneumonia is combined much more frequently with secondary inflammation than with tubercles of different organs; but if in the lungs even tubercles co-exist with disseminated chronic pneumonia, they are much rather its sequence than its cause. Tubercular alterations of other organs may in their turn occasion in the lungs pneumonia foci, either disseminated or confluent. Then, indubitable tubercles of other organs do not permit the decision *a priori*, that the accompanying pulmonary alterations are on that account of a tuberculous nature.

*Acute tuberculization of the lungs, and acute disseminated pneumonia.*—It is only in these acute, or to speak more correctly, subacute affections, that tubercles predominate greatly. I will even add, that very often in disseminated chronic pneumonia, the progress becomes subacute when tubercles have been superadded. Here, then, is the best moment for us to explain what we understand by true tubercles.

The true tubercle is a granulation composed of a cellular proliferation resembling in all respects an inflammatory non-suppurative hyperplasia of connective tissue.

These cellular elements are incompletely developed, surrounded by a solid or gelatiniform intercellular substance; their size varies from one two-hundreth to the one one-hundred and twentieth of a millimetre; their contours are irregular; they exhibit but rarely a distinct nucleus which appears ordinarily closely surrounded with cellular membrane, to such a degree that the membrane of the cell and that of the nucleus form but one body, so to speak. These are the terms in which, since 1844, I have described the cellular element of tubercle, ascribing to the almost solid intercellular substance, in complete development, and I might add cellular aplasia, which causes the granular infiltration to give to the centre of the granulation, at first, and afterwards to the whole of it, a dull yellowish aspect; moreover, there are found sometimes, with these ordinary elements of tubercle, cells more voluminous enclosing these same corpuscles, which I have seen likewise developed in epithelial cells; moreover, I have, since this date, described, as often surrounding true tubercle, granular epithelial cells and pus-cells. I still to-day maintain this to be the correct description of true tubercle. M. L. Meyer,* in a work generally much valued, published about three years ago, describes tubercles in an identical manner twenty years after me. I quote the following passage from his work in

* 1 Virchow's Arch., vol. XXX., p. 62, 1864.

order to enlighten those who have thought proper to attack my description, and of whom several have burlesqued it, in a manner very little in harmony with the serious spirit of my work:

"The characteristic element of the tubercular focus, says M. Meyer, consists in little cells closely juxtaposed, with opaque margins, with simple nuclei as their principal and sole contents, seeing that this nucleus is ordinarily embraced to such a degree by the cellular membrane that the whole cells might be taken for a nucleus. These cells are of unequal size, smaller than those of pus, ordinarily of the size of blood globule, rarely altogether round, etc. Moreover, there are found multinuclear cells belonging in the vicinity, corresponding to an anterior phase of the development, true mother-cells of the little tubercle cells. I am, moreover, satisfied that even those who have attacked my observations have figured within a slight shade, the elements of tubercle as I have. In the last No. of my great work on Pathological Anatomy (1861), I described and figured the development of tubercle as a cellular proliferation of connective tissue.

"In order to show that, from the beginning, in spite of the inferiority of the methods then attainable, I have separated the surrounding inflammatory products from pulmonary tubercle, I should quote, amongst others, the eighteenth aphorism of the conclusion of my work, in the Archives of Müller, of 1844. There is sometimes found around tubercle a particular form of chronic inflammation, with yellow hepatization, of increased consistency. The tissue of the pulmonary vesicles, of the minute bronchial tubes, of the interstitial tissue, is found filled with coagulated fibrine, with fibres of new formation, with granular epithelial cells, and in the midst of these foci of chronic hepatization of slight vascularity, are found foci of acute lobular pneumonia rich in sanguineous vessels."

This represents, moreover, the state of the question in 1843, in which I found nearly a complete blank for microscopic observations, and it must be admitted that it certainly was an advance to offer at that time these descriptions in total opposition to the two reigning opinions, that of the amorphous character, and that of the purulent origin of true tubercle. My principal object, at the epoch, was to demonstrate the organized and cellular nature of true tubercle, and the difference between these cells and those of pus.

Even to this day, I am reproached, first in Germany and later in France, with having regarded these cells as the specific element of tubercle. If such was my interpretation, it was more than twenty years ago, and it has been likewise a considerable number of years since I have professed the conviction that the microscopic elements of tubercle were not the essential part of it, nor even the specific cell. I ask indulgence from no one, and if any one will do me the honor to quote my observations, I have the right to exact that he should be familiar with them, and that he should not assume an obsolete point of departure as fixed and in force with me to-day, when it is so easy to bring proof to the contrary. Each year, and especially since 1856, I have pronounced against the specific character of the cellular elements of tubercle. Let it suffice for me to quote two additional passages from my works, which I believe decisive and irrefutable in this relation:

I. I state in my Pathological Anatomy: * "If I recognize specific elements as the anatomico pathological diagnostic of tubercle, far be from me the thought that these little cells enclose the essence of tubercle and constitute its specific elements. The discussion between my adversaries and me would only involve a difference of opinion of the value of these elements as means of microscoped diagnosis."

II. In 1858,† I declared in the Medical Journal (Nebdomadaire) of Vienna, that morbid products, altogether different from tubercles, exhibited the same little cells, and could not be distinguished from tubercle by the microscope, and upon the occasion of a case of glanders, I pointed out, as microscopically almost identical with tubercle, the little tumors, not

* Anatomie Pathologique, tome 1, page 668.

† Weimer Medicinische Wochenscrift. Jahrgang 111. Page 703.

yet suppurated, of glanders, and recent gummy spyhilitic tumors. I pointed out as the cause of this microscopic similarity a similar mode of formation anatomically. I add that this resemblance of three products so profoundly different in pathology, should furnish a new proof that we should judge a disease from an assemblage of all the characters, and not from an isolated series of signs. I terminated these reflections by remarking that they proved in a very clear and concise manner that in pathological anatomy and in pathology, the microscope might be a useful and faithful servant, but should never become the lord and master. If, after these citations, any one should still apply to me the reproach of sustaining the microscopic specificity of tubercle, it is clear that he expresses by it his own criticism and not mine.

The passage of the cell of the connective tissue in process of proliferation towards tubercle, may be well studied especially upon serous membranes. It is not rare to see there, in the neighborhood of tubercle, disseminated groups of these cells in process of hyperplasia, and they will be found also throughout their progress up to the masses which form the granulations. Moreover, it appears to me more and more probable, that other cells than those of a connective tissue may also give origin to tubercle corpuscles. In the case of miliary granulations, some of them are often seen, with a good lens, much smaller, almost microscopic. It is in the summit of the lungs that their number is the most considerable, whether they be isolated or in groups. With some care, one may see not unfrequently, granulations in the tracheo-bronchial mucous membrane. They are ordinarily yellowish. I have here seen no semi-transparency.

Besides the granulations, there are likewise found in the summits of the lungs, sometimes little inflammatory infiltrations, lobular or a little more extensive grayish or semi-transparent, or little yellowish foci of true disseminated pneumonia foci, either granular or lobular, compact or already softened at the centre, even to pisiform cavities of the volume of a nut or even greater. Even in some cases of acute disease of the lung, with every appearance of true tuberculization; there have been found nothing else than disseminated and confluent pneumonia foci.

If we thus perceive new points of contact between disseminated chronic pneumonia and acute tuberculization multiplied, another fact more important still appears to dominate, so to speak, this one. In the great majority of autopsies of acute tuberculous affections, there are found old foci of disease; amongst these by far the smaller number consist of old tuberculous granulations truly healed, shriveled, surrounded by black pigment. More frequently, on the contrary, there are found in the pulmonary summits old, yellowish pneumonic foci, from the smallest size up to that of a pea or of a bean, mingled with much pigment and callous, and apparently cicatricial connective tissue.

At other times there are found old, yellowish pneumonic foci, which have preceded the appearance of the tubercles by weeks or months. In other cases still, and quite frequent likewise, extensive tuberculous cavities with irregular walls, sinuous, enclose a purulent or muco-purulent detritus, coexistent with the recent tubercles of acute character, as evidence of the ulcerative pneumonia anterior to the granulations. Lastly, the bronchial glands, in subjects who have succumbed to acute tuberculosis, often enclose an old, yellowish infiltration still rich in cells, or a chalky deposit or true stony concretions. All these conditions may be combined in these different modes. I have avoided up to this time giving statistical statements of recorded facts, and reserving these data for a voluminous work upon chronic pneumonia and pulmonary tubercles, upon which I have been for a long time engaged. Nevertheless, I can not resist the temptation of communicating here the following statistical statement:

Without reckoning the more recent facts, not yet analyzed, I possess sixty-six personal observations, with autopsies of acute tuberculization, free or associated therewith by many points of contact. Eleven times only, or in sixteen per cent. nearly, there existed only three tuberculous granulations without trace of old deposits, but it is possible that some may have escaped my notice, since formerly I did not attach nearly so much importance to this coincidence as at present. Six times there were acute tubercles combined with recent disseminated pneumonic foci; once there was general acute tuberculization, the lungs not being involved. There remain but forty-eight cases, or about seventy-five per cent. in which the old foci existed; four times there were tuberculous granulations alone, and in all the other cases pneumonic foci, cavities and old infiltrations of bronchial glands. It is very natural to seek in this coincidence the relation of cause and effect, and just as in disseminated chronic pneumonia, complicated with tubercles, these are consecutive, so likewise acute tuberculization is, in a sufficiently large majority of cases, consecutive to anterior pneumonia foci. We are struck with this other fact that acute tuberculization has been preceded by anterior miliary tubercle, infinitely more rarely than by inflammatory foci.

Another fact under our observation comes to the aid of the influence of prolonged anterior inflammatory process upon the production of tubercles. There are two of our observations in which a chronic purulent pleuritic effusion preceded, and probably occasioned, an acute consecutive pulmonary tuberculous affection.

Let us observe, in passing, that the four cases of old healed tuberculous granulations are evidence in favor of the curability of acute tuberculization, and serve to corroborate my diagnosis in two cases, in which I saw patients exhibiting every evidence of acute tuberculization of the lungs, gradually restored to health.

As to the seat of these tuberculous granulations, to judge by the naked eye, the interstitial tissue, the air cells, the bronchial ramifications, the subpleural tissue, and the pleura appear to be attacked. It is especially important to fix their point of departure and origin. In the pia mater this takes place along the vessels, and in the lymphatic fluid, which surrounds the arterioles. According to Virchow, it is less the vessels than the fundamental tissue of organs which would give origin to tubercles. Lelleyer has observed the same in the non vascular organs in the corpuscles of Pacchioni. My histogenetic investigations have not, thus far, led me to satisfactory results; however, I desire henceforward to put myself on the defensive against every exclusive theory upon this subject. Colberg, in his excellent *investigations into the formation of tubercles*, admits that at a later epoch, the cellular tissue and the sanguineous vessels are effected with tubercular proliferation, just as alveolar inflammation originally epithelial may involve the interstitial tissue and the blood-vessels, but he indicates the cells of the capillary blood-vessel as the constant and exclusive point of departure of true tubercle.

I do not question the accuracy of these observations; whilst I can not yet decide upon so exclusive a genesis of tubercle by the capillary cells alone. We see in the heart the vascular structure in the endocardium with all its layers, a powerful muscular mass, to which is added the apparatus of a force pump of great power; then we perceive the elastic and fibrocellular elements of the arteries diminish perceptibly in the veins.

Lastly, in the capillary vessels there remains no more than a single membrane, the internal epithelial tunic.

This important fact has been demonstrated in a certain manner by the beautiful investigations of Dr. Auerbach (of Breslau) and he calls these cells — which, reunited, form the capillary tube — perithelia; it is clear that these cells placed together and reunited, in order to form in their continuity a smooth and continuous tube of uniform calibre, could not become the seat of a true proliferation unless the capillary vessel might disappear; the blood would find itself then in the lacunæ, in place of circulating in the canals. Then the proliferation could only originate from the nuclei of these perithelial cells, and here especially would the capillary ruptures be frequent in consequence of this hyperplasia.

I have even, during a long scientific career, willingly conceded that theoretical objections ought to yield to observation, especially emanating from a source so good. In admitting, then, that the perithelia of the capillary vessels serve as an essential point of departure for tubercle, we will with difficulty comprehend that these cells having so great an affinity to epithelia, these latter should be excluded from all participation in the tubercular endogenesis. I would add, that in my experiments, pulmonary tubercles, established in animals by inoculation of human tubercles, have appeared to have especially for their base cells very like epithelia.

On the other hand, I admit likewise willingly that even the yellow alveolar and epithelial miliary granulations of the lungs are completely different from true tubercles, and I have been able, in miliary and lobular disseminated pneumonia, without subacute tubercles, to follow all the phases of alveolar epithelial proliferation with the most extensive inflammatory foci in which the yellow infiltration was combined with diffuse gemmation of conjunctive tissue, colored by an abundant pigment, and in one case, which at other times would have been assimilated to acute tuberculization, I have seen these confluent foci as large as an apple, and even, in one of the lobes, equaling two thirds of its contents.

The pulmonary tissue around true granular tubercles appears very often entirely healthy, even anæmic, in consequence of a species of compensatory emphysema. At other times one finds around all its phases a simple hyperæmia, with alveolar collapse, or even little inflammatory infiltrations. Moreover, there is perceptible in the infero-posterior portions of the lungs, quite frequently, carnification and emphysema upon the pulmonary borders, as well as in the anterior and superior parts. Pleuritic adhesions are frequent, and the pleura offers, besides the microscopic granulations, groups of fusiform conjunctive cells in process of proliferation, and likewise little circumscribed thickenings of the sub-pleural tissue, exhibiting in an especial manner the appearance of drops of wax. These, then, are the irregular points of cellular multiplication, as in the inflammation, accompanying true tubercle.

In one-fourth of the cases, I have found pleuritic effusion from half a quart to a quart and a half of serous or sero-purulent fluid.

In two cases in which tuberculous granulations were developed subsequently to the pleuritic effusion, this latter, chronic and purulent, has appeared to be the point of departure. A fact which corroborates this view of the question has been communicated to me recently by Dr. Ebstein, the distinguished pathological prosecutor of the hospital of Breslau. A woman suffering from organic disease of the heart with very serious mitral insufficiency, was attacked by caries of the femur with abundant and prolonged suppuration, and fell a victim to acute tuberculization of the lungs, a disease most rare in the course of an organic disease of the heart. Legendre,* in reference to tuberculous meningitis, reports that, in this disease, twenty-seven out of twenty-eight cases exhibited very numerous tuberculous granulations; in twenty-four cases a tuberculous infiltration much older, extensive and yellowish existed in the bronchial glands, as has occurred hitherto under our observation, old foci having preceded without having occasioned, by infection, acute tuberculous disease. I have been struck with the relative frequency of granulations of the pericardium with acute pericarditis in the course of acute tuberculosis. The heart ordinarily encloses clots, some soft, others fibrinous. Obstruction of the large veins is rare, I have observed it in the crural and in the jugular. Tuberculous meningitis in acute tuberculization is much more frequently found in the adult, according to my observation, than is generally believed. It is, as during infancy, accompanied by internal hydrocephalus, by plastic meningitis of the base and sometimes by more voluminous yellow tubercles of the brain.

Tubercles are rare in the stomach, more frequent in the small intestine, which may nevertheless exhibit non tuberculous ulcers.

As in infectious diseases, the intestinal glands and the spleen are often perceptibly tumified, even without trace of

* Recherches sur quelque maladies de l'enfance, Paris, 1846, p. 12.

tubercles. The mesenteric and retro-peritoneal lymphatic glands have been, under my observation, quite frequently the seat of old yellow tuberculiform infiltrations long anterior to the acute tuberculous affection. The peritoneum is frequently the seat of true tubercles, with or without peritonitis, but there are found here likewise simple cellular masses and granulations, more decidedly inflammatory. Colberg has determined very clearly in the epiploon likewise the formation of tubercles by hyperplasia of the nuclei of the capillary vessels.

In acute tuberculosis the liver quite frequently encloses granulations, and moreover many very small, which can be determined by examination with a lens. No one has better described their formation than E. Wagner.* According to him, the hepatic cells are at first seen interrupted at intervals by masses of nuclei, sometimes indeed quite separated by microscopic tubercles. The nuclei of the tubercles take their origin in the cells of the interstitial connective tissue, or from the external tunic of the hepatic vessels, or finally from the nuclei of the investment of the hepatic cells, which indeed play the principal part in their formation.

The multiplication of these nuclei takes place very rapidly, partly by division, partly by new formation, as this author still admits, although it is perhaps at this time generally denied. Moreover, men of the highest merit, like Robin and Broca, admit it likewise, and I confess that whilst admitting cellular division and endogenesis as the principal sources of their pathological formation, it is impossible, notwithstanding the most attentive study, always to arrive at the determination of this mode of formation of morbid products. Further, if cells can be formed freely by endogenesis on the interior of other cells, I see no reason why the same phenomena might not occur outside of cellular membranes by a species of exogenesis. Besides it is better here, as well as for generation generally, to admit, wherever generic observation fails, an unknown genesis in addition to that by proliferation, and that

* Dir tuberculose der leber (Archerder Heilkunde, von Roser, Wunderlich, eter.)

by endogenesis, as to make use, without sufficient proof, of the term spontaneous generation.

The spleen, moreover, whilst it is often diseased in acute tuberculosis without granular deposits, still quite frequently contains true miliary tubercles.

The same is true of the kidneys, in which, however, tubercles, still very small, have ordinarily a yellow color. Parenchymatous nephritis is rare in the disease now under consideration.

In cases of complication of acute tubercle with those of the testicle, which I have observed, this latter organ contains them ordinarily much older, and still more frequently infiltrations of a yellowish appearance and inflammatory origin, than granulations.

I have likewise, also, observed several cases in which acute pulmonary tuberculization supervened very rapidly in the course of an old chronic tuberculous, orchitis, or epididymitis.

The thyroid gland has presented to me several times true tubercles. In Switzerland I have observed all the forms of goitre amongst patients who had sunk under tuberculous disease, and I cannot comprehend how any one can admit an antagonism between goitre and tuberculosis. I terminate this sketch of true tubercle by insisting upon the fact, that it is almost always multiple, not only in one but in a certain number of organs, multiplicity being so much the more generalized as the subject is young and approximating infancy.

